# Repeated Fecal Microbial Transplantations and Antibiotic Pre-Treatment Are Linked to Improved Clinical Response and Remission in Inflammatory Bowel Disease: A Systematic Review and Pooled Proportion Meta-Analysis

**DOI:** 10.3390/jcm10050959

**Published:** 2021-03-01

**Authors:** Valentin Mocanu, Sabitha Rajaruban, Jerry Dang, Janice Y. Kung, Edward C. Deehan, Karen L. Madsen

**Affiliations:** 1 Department of Surgery, University of Alberta Hospital, University of Alberta, 8440 112 Street NW, Edmonton, AB T6G 2B7, Canada; dang2@ualberta.ca; 2 Division of Gastroenterology, Department of Medicine, University of Alberta, Edmonton, AB T6G 2E1, Canada; rajaruba@ualberta.ca (S.R.); deehan@ualberta.ca (E.C.D.); kmadsen@ualberta.ca (K.L.M.); 3 John W. Scott Health Sciences Library, University of Alberta, 2K3.28 Walter C. Mackenzie Health Sciences Centre, Edmonton, AB T6G 2R7, Canada; Janice.kung@ualberta.ca

**Keywords:** inflammatory bowel disease, fecal microbial transplantation, antibiotic treatment

## Abstract

The response of patients with inflammatory bowel disease (IBD) to fecal microbial transplantation (FMT) has been inconsistent possibly due to variable engraftment of donor microbiota. This failure to engraft has resulted in the use of several different strategies to attempt optimization of the recipient microbiota following FMT. The purpose of our study was to evaluate the effects of two distinct microbial strategies—antibiotic pre-treatment and repeated FMT dosing—on IBD outcomes. A systematic literature review was designed and implemented in accordance with the Preferred Reporting Items for Systematic Reviews and Meta-Analyses (PRISMA) guidelines. A medical librarian conducted comprehensive searches in MEDLINE, Embase, Scopus, Web of Science Core Collection, and Cochrane Library on 25 November 2019 and updated on 29 January 2021. Primary outcomes of interest included comparing relapse and remission rates in patients with IBD for a single FMT dose, repeated FMT dosages, and antibiotic pre-treatment groups. Twenty-eight articles (six randomized trials, 20 cohort trials, two case series) containing 976 patients were identified. Meta-analysis revealed that both repeated FMT and antibiotic pre-treatment strategies demonstrated improvements in pooled response and remission rates. These clinical improvements were associated with increases in fecal microbiota richness and α-diversity, as well as the enrichment of several short-chain fatty acid (SCFA)-producing anaerobes including *Bifidobacterium*, *Roseburia*, *Lachnospiraceae*, *Prevotella*, *Ruminococcus*, and *Clostridium* related species.

## 1. Introduction

Inflammatory bowel disease (IBD) is a chronic inflammatory condition of the gastrointestinal tract categorized by Crohn’s disease (CD), ulcerative colitis (UC), and indeterminate colitis [1,2]. The incidence of IBD is steadily increasing worldwide [3], as are its extensive healthcare and economic burdens. While IBD is believed to involve a host’s genetic predisposition, environmental factors, and an imbalanced gut microbial community, the etiology of IBD has yet to be fully elucidated [4,5,6,7,8]. The complex pathophysiology underlying IBD has led to the current implementation of non-specific therapeutic strategies centered on systemic immunosuppression [9,10]. Despite the significant complications associated with these strategies, ongoing high rates of refractory disease remain [11,12,13] suggesting that alternative targeted approaches are needed to enhance the clinical efficacy and safety of modern IBD therapies [14].

Accumulating evidence suggests that imbalances in the gut microbiome, a highly diverse community of microorganisms that inhabits the gastrointestinal tract of humans, plays a causative role in the pathogenesis of IBD [15,16,17]. In general, gut microbial communities of patients with IBD are characterized by reduced microbial diversity, an increased abundance of aerobic pro-inflammatory bacteria, and a reduction in anaerobic bacteria that generate beneficial anti-inflammatory metabolites, such as short-chain fatty acids (SCFA). These findings have fostered growing interest in adopting microbiota-targeted strategies into the forefront of modern IBD therapeutics [18,19,20] in order to reduce the need for long-term immunosuppressants and their associated adverse complications.

Fecal microbial transplantation (FMT) is one such microbiota-targeted strategy that has shown initial promise for the management of IBD by implanting members of microbiota from healthy donors in an attempt to restore imbalances in host-microbial ecology [21]. However, clinical response of IBD to FMT has shown extensive inter-study heterogeneity [22], which might stem from the variable engraftment of donor derived microbes and the high or persistent populations of unfavorable pathobionts in the host [23,24,25,26]. In this regard, both antibiotic pre-treatments (to lessen competitive interactions) and increased frequency of FMT delivery may both enhance the engraftment of putatively beneficial microbes, correcting dysbiotic populations, and promoting clinical response and disease remission [27,28,29,30]. While several trials utilizing either antibiotic pre-treatments [31,32,33,34] or repeated FMT regimens [35,36] have been conducted in patients with IBD, no pooled analyses of these findings exist, therefore hindering the optimization of FMT-based IBD therapies.

The purpose of our study was to address this important gap in knowledge by conducting a systematic review and meta-analysis to characterize the effects of antibiotic pre-treatment and repeated FMT dosing on IBD response and remission. Our primary outcome was to compare differences in pooled relapse and remission rates between antibiotic pre-treatment and repeated FMT dosing strategies. Secondary outcomes included comparing differences in fecal microbiota composition associated with disease response and remission for these two approaches.

## 2. Methods

### 2.1. Eligibility Criteria

A systematic literature search strategy was designed using the Population, Intervention, Comparison, Outcome, and Study Design (PICOS) framework and implemented in accordance with the Preferred Reporting Items for Systematic Reviews and Meta-Analyses (PRISMA) guidelines. FMT was defined as the administration of a fecal matter solution from a healthy donor to the gastrointestinal tract of a recipient to confer a health benefit. Our inclusion criteria included studies with adults (age ≥ 18 years) that had a diagnosis for IBD and received FMT. All modalities of FMT delivery, such as colonoscopy, nasogastric tube, oral capsules, or enemas, and any regimen of antibiotic pre-treatment were included. Studies were excluded if disease was localized to the surgical pouch (i.e., pouchitis), patients had concurrent *Clostridioides difficile* infection, less than six patients were enrolled, or in a pediatric population. Duplicate studies, kin studies, studies using animal models, and non-English studies were also excluded.

### 2.2. Search Strategy

A medical librarian (JK) systematically searched the MEDLINE (via Ovid), Embase (Ovid), Scopus, Web of Science Core Collection, and Cochrane Library (via Wiley) databases on 25 November 2019 (see Supplemental Table S1 for full-text search strategy) and updated on 29 January 2021. No language or date limits were applied. To complement this approach, the research team also screened the first 200 results from Google Scholar for inclusion. Manual searches of references from included studies were further performed to identify potentially missed articles.

### 2.3. Study Selection

Titles and abstracts of relevant articles were first manually screened for inclusion by two independent reviewers (VM, SR). Studies meeting initial screening criteria by at least one reviewer were selected for a full text review by two independent reviewers (VM, SR) using pre-specified inclusion and exclusion criteria. Disputes were resolved by a third reviewer (JD). Data were extracted independently by two reviewers (VM, SR) into separate Excel spreadsheets and cross-examined for accuracy. Studies were then assessed for methodological quality and bias using the Newcastle-Ottawa [37] tool for cohort studies and the Cochrane Risk of Bias [38] evaluation for randomized controlled trials (RCTs).

### 2.4. Data Extraction

Study characteristics were evaluated for study design, year, and country of origin. Primary outcomes of interest included relapse and remission rates following FMT. Secondary outcomes included differences in fecal microbiota composition, and adverse events. Patient characteristics included age, sex, mean disease duration, type of IBD, histology disease scoring, and current medications. FMT strategy-specific variables included donor stool processing, mode of delivery, type of FMT regimen, and type and duration of antibiotic pre-treatments.

### 2.5. Data Synthesis

Continuous data were expressed as mean ± standard deviation (SD). For the purpose of meta-analysis, data extracted as medians and interquartile ranges were converted to mean ± SD using methods outlined by Hozo et al. [39]. Meta-analyses of pooled proportions were conducted using a random effects models by the DerSimonian-Laird method [40]. Estimates of heterogeneity were obtained from inverse-variance fixed-effect models. Pooled estimate variances were stabilized using the Freeman-Tukey Double Arcsine Transformation. Heterogeneity was assessed using the Chi-squared test with significance set at *p* < 0.10 and the amount of heterogeneity quantified by the I^2^ statistic as low <50%, moderate 50–75%, or high >75% [41]. Categorical data were assessed using either Chi-squared or Fischer’s exact tests. A two-sided α of less than 0.05 was considered statistically significant. Meta-analysis was conducted using the metaprop function in STATA (v15.1; StataCorp, College Station, TX, USA).

## 3. Results

### 3.1. Search Results and Study Characteristics

**Figure 1:**
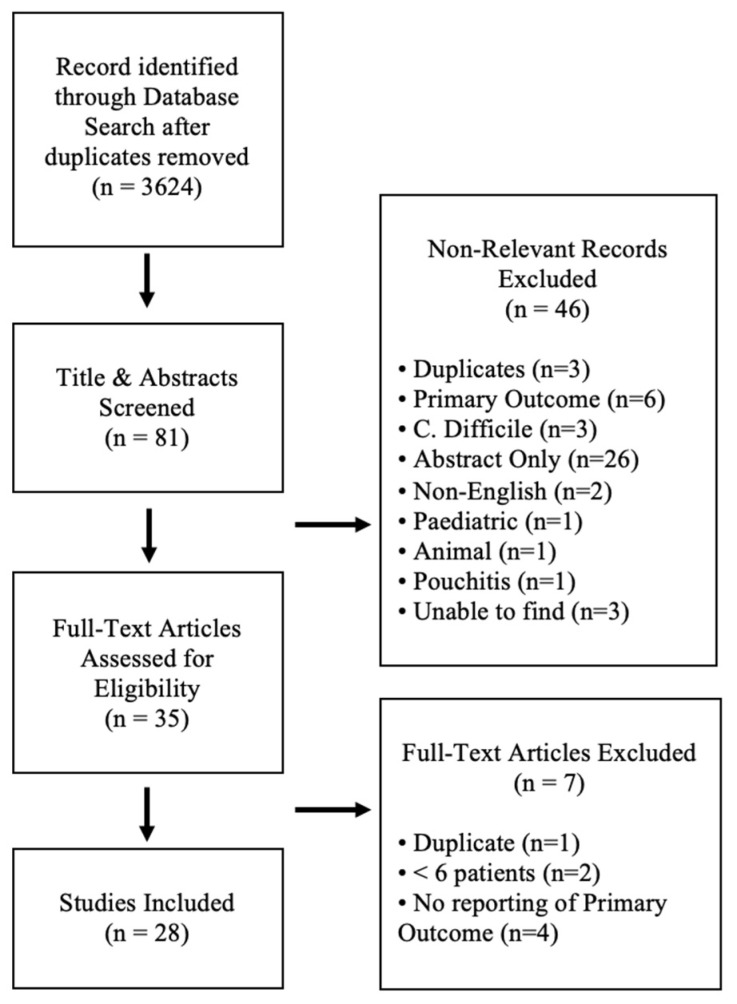
PRISMA flow chart of assessed studies.

Comprehensive search of the five databases yielded a total of 4220 results, and after duplicate records were removed, 3624 articles remained (Figure 1). After initial screening of the titles and abstracts, the text of 45 articles were fully reviewed. Following full text review, 28 articles were eligible for inclusion in the final systematic review. No prior systematic reviews examining FMT outcomes with respect to antibiotic pre-treatment or repeated FMT regimens were identified. Of the included articles, six were randomized controlled trials, 20 were prospective cohort trials, and two studies were case series.

Of the 28 studies reviewed, 22 included patients with UC, four included patients with CD, and two studies assessing both UC and CD. Most studies examined disease response in patients with mild to moderate disease (*n* = 9 studies), with twelve studies assessing patients with severe disease (Table 1). Study duration and follow-up ranged from 4 weeks to 13 years with most studies having a follow up ≤12 weeks (*n* = 17). Five studies utilized pre-operative antibiotics prior to FMT, with only two studies utilizing the same antibiotic regiments. Nearly half of the studies included a single FMT delivery (*n* = 12), while the remaining trials use varied regimens.

### 3.2. Risk of Bias Assessment

Risk of bias for cohort studies was characterized using an adjusted 7-point Newcastle-Ottawa scale of selection, comparability, and study outcome categories (Supplemental Table S2). The 19 included cohort studies demonstrated low to moderate risk of bias due to a lack of long-term follow-up greater than three months (*n* = 7 studies), and inadequate description or evaluation fecal microbiota changes (*n* = 8 studies). The six randomized trials were assessed for bias using the Cochrane Risk of Bias tool and together demonstrated low risk of bias (Supplemental Table S3).

### 3.3. Baseline Demographics

A total of 976 patients were identified from the 28 studies included (Table 2). Twenty-two studies included only patients with UC (*n* = 767), while three studies included patients with CD (*n* = 87) alone. The mean weighted age of all patients was 40.0 years, of which 59% were on average male with a mean weighted disease duration of 6.2 years. The proportion of patients receiving concurrent corticosteroids varied extensively from 7% to 100%. Patients with a diverse spectrum of IBD severity were included although the majority of included patients had mild-moderate disease (*n* = 439; 9 studies). Prior to FMT, total Mayo scores for UC activity ranged from 6.1 to 11.1 and CD activity index ranged from 275 to 345. No significant differences in clinical characteristics were observed between CD and UC patients prior to FMT.

### 3.4. FMT Administration, Dosing, and Donor Characterization

FMT methodologies varied substantially across all studies. The most frequent mode of FMT was via colonoscopy (*n* = 19 studies), followed by nasoduodenal/naso-jejunal tube (*n* = 4 studies), enemas (*n* = 4 studies), gastroscopy (*n* = 3 studies), and oral capsules (*n* = 1 study). The dosage of FMT ranged from 12 g to 300 g of stool per administration with 50% (*n* = 10 studies) of all studies delivering multiple doses. Antibiotic pre-treatment regimens ranged from three to 14 days prior to FMT (*n* = 5 studies), with most studies using a combination of antibiotics (*n* = 4 studies) and specifically vancomycin (*n* = 3 studies). FMT donors of included studies were typically healthy donors unrelated to the recipients. Nine studies utilized donors that were either relatives or specifically chosen by the patients.

### 3.5. Response and Remission Rates for Repeated FMT Regimens

**Figure 2:**
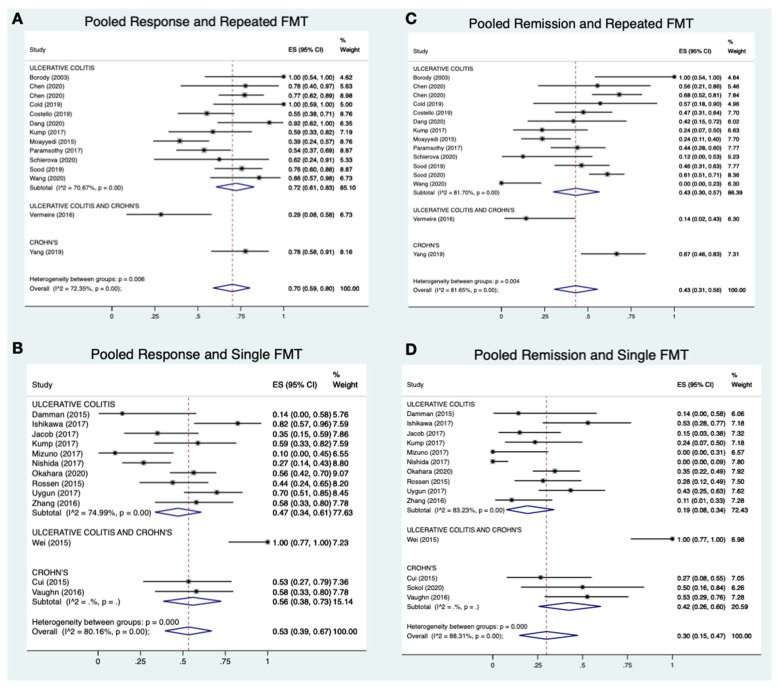
A-2D: Meta-analysis of pooled response and remission rate for repeated vs. single FMT.

Of the 976 patients included, 41.9% (*n* = 409) were treated with a single FMT and 30.0% (*n* = 229) with repeated FMT (Table 1 and Table 3). Meta-analysis of all included studies revealed that repeated FMT studies had higher pooled response rates (15 studies; 70%; 95% CI 59–80%; I^2^ = 72%; Figure 2A) than those with single FMT (13 studies; 53%; 95% CI 39–67%; I^2^ = 80%; Figure 2B). Pooled remission rates for studies with multiple FMTs (15 studies; 43%; 95% CI 31–56%; I^2^ = 82%; Figure 2C) were also higher than for studies with a single FMT (13 studies; 30%; 95% CI 15–47%; I^2^ = 88%; Figure 2D).

Subgroup analysis of UC studies revealed more pronounced differences in pooled response (12 studies; 72%; 95% CI 61–83%; I^2^ = 71% vs. 10 studies; 47%; 95% CI 34–61%; I^2^ = 75%) and remission rates (12 studies; 43%; 95%CI 30–57%; I^2^ = 82% vs. 10 studies; 19%; 95% CI 8–34%; I^2^ = 83%) when comparing repeated and single FMT regimens, respectively.

Taken together, pooled response and remission rates were more favorable for patients receiving repeated FMT regimens than single FMT alone. Heterogeneity for all pooled analyses was high with all I^2^ values greater than 70%.

### 3.6. Response and Remission Rates for Antibiotic Pre-Treatments

**Figure 3:**
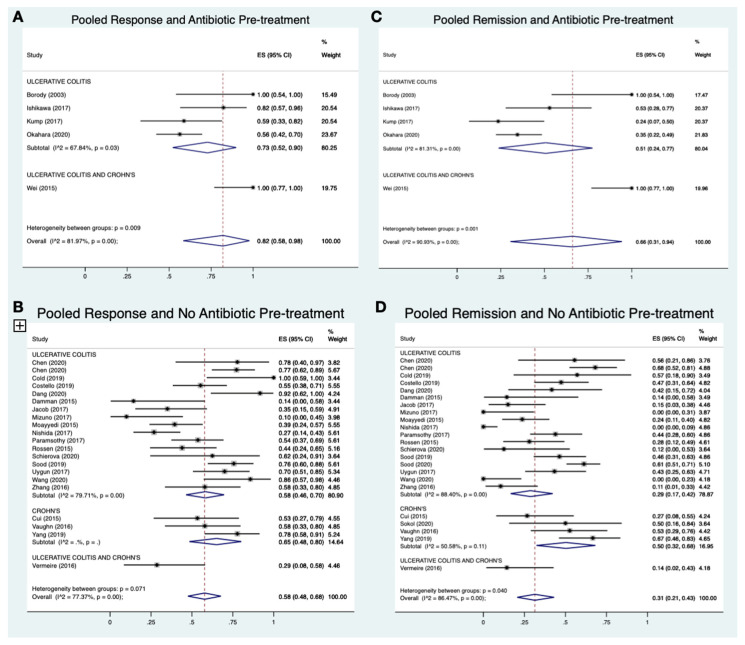
A-3D: Meta-analysis of pooled response and remission rate for antibiotic pre-treatment vs. no pre-treatment.

Antibiotics were not frequently administered as pre-treatments, with only 11.2% (*n* = 109) of patients receiving an antibiotic regimen prior to FMT. Meta-analysis of included studies revealed that pooled response rates for antibiotic pre-treatment (five studies; 82%; 95% CI 58–98%; I^2^ = 82%; Figure 3A) were higher than for no pre-treatment (23 studies; 58%; 95% CI 48–68%; I^2^ = 77%; Figure 3B). Likewise, antibiotic pre-treatment was also associated with improved remission rates (five studies; 66%; 95%CI 31–94%; I^2^ = 91%; Figure 3C) when compared to no pre-treatment (23 studies; 31%; 95%CI 21–43%; I^2^ = 86%; Figure 3D).

The favorable effect of antibiotic pre-treatment on pooled response (four studies; 73%; 95% CI 52–90%; I^2^ = 68% vs. 17 studies; 58%; 95% CI 48–70%; I^2^ = 80%) and remission rates (four studies; 51%; 95% CI 24–77%; I^2^ = 81% vs. 18 studies; 29%; 95% CI 17–42%; I^2^ = 88%) was also observed on subgroup analysis of UC studies.

Similar to the repeated FMT analysis, heterogeneity for the pooled proportion analyses of antibiotic pre-treatment was high.

### 3.7. Fecal Microbiota Compositional Changes Following FMT

#### 3.7.1. Overview of Microbiota Reporting of Included Studies

Although FMT aims to shift the gut microbial communities of patients with IBD, only 64% of studies (*n* = 18 studies) characterized the recipient’s fecal microbiota following FMT and only two studies directly assessed associations between IBD remission and fecal microbiota compositional changes (Table 4). Further, no study directly compared microbial changes of antibiotic pre-treatment vs. no pre-treatment or repeated FMT vs. single-dose FMT. Only five studies provided donor microbial characterization. The majority of studies (*n* = 14 studies) used 16 s rRNA gene amplicon sequencing methods, with three studies using whole-genome sequencing and one using *Bacteroides* HSP60 sequencing.

#### 3.7.2. Changes in Alpha and Beta Diversity Following FMT

Of these 18 studies, nine (50%) reported an increase in microbial richness and α-diversity following FMT, as estimated by the abundance of operational taxonomic units (OTUs), Chao1, Simpson and Shannon indices. Six studies reported no change in α-diversity after FMT. Changes in β-diversity evaluated using Bray-Curtis dissimilarity were reported in five studies, with the majority (*n* = 4 studies) showing that the microbial ecology of FMT recipients underwent shifts towards those of their respective donors. Within these four studies, increased engraftment was associated with improved clinical outcomes.

In terms of specific bacterial shifts, the effects of FMT were shown to be highly variable (Table 4). Nonetheless, 15 of the 18 studies (83%) that evaluated for shifts in specific gut microbial taxa reported increases in the abundance of anaerobes purported to produce health promoting anti-inflammatory SCFAs, such as *Bifidobacterium*, *Roseburia*, *Lachnospiraceae*, *Prevotella*, *Ruminococcus*, and *Clostridium* related species.

#### 3.7.3. Recipient and Donor Microbial Ecology Associated with IBD Outcomes

Findings from the two studies that assessed associations between IBD remission and fecal microbiota compositional and functional changes were also variable. Parmsothy et al. provided the best assessment of bacterial taxa and corresponding metabolic pathways related to specific IBD outcomes. Following intensive multi-donor FMT, patients with sustained remission had increased relative abundance of *Eubacterium halii*, *Roseburia inulivorans*, and *Ruminococcus* while those who relapsed had higher proportions of *Fusobacterium*, *Escherichia*, and *Prevotella.* Metabolomics of remission patients further revealed increased activation of metabolic pathways associated with the biosynthesis of SCFAs and secondary bile acids. In addition, only one study by Kump and colleagues explored the role of donor microbiota with respect to IBD outcomes following FMT. Patients that received donor fecal microbiota of greater bacterial richness and α-diversity (assessed by OTU abundance and Shannon diversity) and with increased *Ruminococcus* and *Akkermansia* abundances were shown to have higher rates of IBD remission.

### 3.8. Reported Adverse Events

Overall, FMT in patients with IBD was shown to be safe and well tolerated. Frequently reported symptoms related to FMT included a transient self-limiting fever alleviated with paracetamol, and non-specific transient gastrointestinal symptoms such as abdominal discomfort, bloating, nausea, vomiting, and diarrhea (Table 5). Of 26 studies that reported serious adverse events, 13 patients with UC required colectomies and one required hospitalization due to disease progression. One patient also contracted *Clostridioides difficile* requiring a colectomy and one patient contracted cytomegalovirus infection seven weeks after FMT. Overall, the reported serious adverse events were suggested by the authors to be unrelated to the FMT therapy. No patient receiving FMT intervention in the included studies suffered mortality.

## 4. Discussion

To our knowledge, we present the first systematic review and meta-analysis evaluating the effects of antibiotic pre-treatment and repeated FMT approaches on improving response in patients with IBD response. Notably, our meta-analysis revealed that repeated FMT and antibiotic pre-treatment were associated with improvements in both pooled IBD response and pooled remission rates. These improvements were associated with key changes in fecal microbial composition such as increased bacterial richness, α-diversity and relative abundance of anaerobes purported to produce SCFAs. Taken together, our findings are novel in that they highlight the potential of these microbiota-targeted strategies to optimize the efficacy of FMT for the management of IBD.

Our findings are in agreement with previous systematic reviews and meta-analyses examining the impact of FMT as a therapy for IBD. In 2014, Colman et al. first identified a lack of literature characterizing FMT treatment efficacy despite publications investigating FMT therapy for IBD more than doubling since 2012 [64]. The systematic review and meta-analysis of 18 studies consisting of 122 IBD patients by Colman and colleagues further revealed that the pooled proportion of patients achieving clinical remission was 36.2% (95% CI 17.4–60.4%). The authors concluded that, while FMT demonstrated variable efficacy, further rigorously designed RCTs were needed to determine efficacy, with a particular need for studies that investigate the effects of FMT frequency and route of administration. More recently, Imdad et al. conducted a 2018 Cochrane review examining FMT therapy on IBD response and remission [65]. Four studies with a total of 277 UC patients were identified and revealed an improved clinical response (RR 1.70; 95% CI 0.98–2.95) and endoscopic remission (RR 2.96; 95% CI 1.60–5.48) for patients receiving FMT vs. placebo. These systematic reviews were, however, limited by a lack of high-quality RCTs and standardized fecal microbiota analysis. Our study addresses a number of these gaps by evaluating both high-quality RCTs and cohort studies, which allowed us to specifically characterize the impact of FMT frequency and antibiotic pre-treatment on IBD outcomes.

Repeated FMT strategies have been employed with variable success in a number of different clinical entities thought to be associated with imbalances in host-microbial ecology [67,68,69]. Perhaps the most compelling evidence for repeated FMT is observed in the *Clostridiodes difficile* infection (CDI) literature. In a recent systematic review and meta-analysis by Baunwall et al., repeated FMT was found to be superior to single-dose FMT in management of recurrent CDI (91% vs. 84%) [69]. Similarly, El-Salhy et al. demonstrated an increased clinical efficacy for repeated FMT dosing in patients with irritable bowel syndrome, albeit in a small case series of 10 patients [68]. Lastly, in a double-blinded placebo-controlled pilot trial, repeated FMT in patients with obesity and metabolic syndrome demonstrated successful engraftment of donor derived microbes, but without any clinical improvements in host metabolic parameters [67]. These inconsistencies are in large part due to the dramatic study heterogeneity with respect to donor selection, FMT preparation and route of delivery, as well as underlying differences in host-gut microbiome interactions implicated in disease pathophysiology [70]. Notwithstanding, our study findings indeed suggest that repeated FMT dosing provides a promising approach to improve IBD outcomes by facilitating donor microbe engraftment, increase α-diversity, and promote SCFA producing taxa.

Ongoing debate exists regarding the pre-treatment of recipients with antibiotics prior to FMT to increase efficacy [71,72]. Conceptually, antibiotic pre-treatment helps provides a proverbial ecological clean slate for the engraftment of donor microbes by freeing up otherwise occupied niches. Elegant work by Ji et al. compared antibiotic pre-treatment versus bowel cleansing or no pre-treatment in mice prior to FMT. The authors demonstrated that FMT efficacy was dependent on the number of niches available for donor microbe engraftment [73]. Further, they found that antibiotic pre-treatment proved to be the most effective strategy for enhancing host gut microbiota reprogramming by increasing donor microbe colonization. Work by Freitag et al., on the other hand, demonstrated that antibiotic pretreatment prior to FMT in mice had only minor effects on overall donor microbial engraftment [71]. Antibiotics disrupted pre-FMT host microbial communities, yet only select donor-derived bacterial taxa such as *Bifidobacterium* were increased and no improvements in overall similarity to the donor microbiota were noted. Indeed, questions remain regarding the optimal antibiotic regimens required to make niches accessible, which niches should be targeted for FMT re-colonization, and whether the potential benefit surpasses the potential harm associated with antibiotic resistance and CDI. While our findings are promising as they show improvements in IBD remission and relapse for groups receiving antibiotic pre-treatment prior to FMT, further studies are needed that evaluate the mechanisms and implications of similar approach on IBD.

We acknowledge that our systematic review and meta-analysis has a number of important limitations. Pooled analysis of our primary outcomes demonstrated a high degree of heterogeneity and does not allow for direct comparison of effect size associated with either repeated FMT or antibiotic pre-treatment regiments. The heterogeneity of our results was extensive and, in a large part, due to differences in study design, FMT regimens and individualized responses to FMT. In general, the administration and preparation of FMT is not standardized with practice patterns varying dramatically. Major differences in route of delivery, donor selection, dosing rationale, and antibiotic pre-treatment regimen are all likely to promote inter-study heterogeneity in our review. Follow-up timeframes also ranged from two weeks to 13 years, with nearly half of the studies having a follow up <3 months. This may have introduced a bias towards more favorable clinical response and remission rates following FMT therapy. Therefore, arguments can be made that, given the immense variability of such disparate study interventions, more focused inclusion criteria are warranted in future studies. As this is the first IBD review to evaluate repeated FMT and antibiotic pretreatment concepts, we elected a priori to broadly include all potentially relevant literature in order to highlight current limitations and to allow for explorative hypothesis generation.

Correlations regarding outcomes and antibiotic pre-treatment should also be interpreted with caution given the small proportion of patients within included studies and the lack of direct comparison with patients receiving FMT alone. Histologic assessments pre- and post- FMT were also not consistently reported across studies hindering our ability to evaluate the histologic effects of FMT on disease activity, or the effects of FMT on mucosal adherent bacterial communities. The findings of our review also heavily favored patients with UC and are therefore less generalizable to CD. Additionally, consistent reporting and analysis of fecal microbiota compositional data for both donors and patients were not reported across all studies, which limits the ability to elucidate potential underlying features of the gut microbiome important for optimizing clinical efficacy. Finally, our literature search revealed a number of abstracts and protocols not ultimately published as final manuscripts, which is indicative of publication bias in the FMT literature.

Despite these limitations, our study provides the first systematic review and meta-analysis that evaluates the impact of two key microbial-based strategies which optimize the efficacy of FMT on IBD outcomes. Results of this study may have a number of important implications. Firstly, we demonstrate that repeated FMT dosing and antibiotic pre-treatment approaches have a promising role in optimizing IBD remission and response rates following FMT. Second, results of this study also highlight a need for standardization of FMT therapy protocols (donor, dose, delivery, and pre-treatment) and reporting of microbial data as the lack of this data seen in current practices preclude meaningful meta-analysis of microbial ecology. Lastly, additional high quality randomized trials are needed which directly compare these two strategies in order to help overcome the high degree of heterogeneity in present studies and to elucidate the mechanisms through which these improved outcomes occur. Only through such standardization practices can we eventually bring tailored microbial transplant therapies from the forefront of current IBD research to standard clinical practice.

## 5. Conclusions

Repeated fecal microbial transplantation and antibiotic pre-treatment engraftment strategies in patients with IBD were associated with improvements in pooled response and remission rates following FMT. These improvements were associated with an increase in fecal microbiota richness, α-diversity, and several SCFA-producing anaerobic taxa. Further standardization of FMT therapies is required to bring microbial-targeted therapies based on FMT from the forefront of current IBD research to modern clinical practice.

## Figures and Tables

**Table 1:** Study design and FMT regimen characteristics.

Study	Study Design	Patients (*n*)	Country	Disease	Severity	FMT Delivery	FMT Donor	FMT Dosage	FMT Frequency	Pre-Treatment Antibiotics	Antibiotic Frequency	Total Follow-up (Weeks)
Borody 2003 [31]	Case series	6	Australia	UC	Severe	Enema	Healthy donors chosen by patient	200–300 g/200–300 mL saline	Daily for 5 days	Vancomycin (500 mg bid), metronidazole (400 mg bid), rifampicin (150 mg bid)	7–10 days	676
Chen 2020 [42]	Prospective cohort	9	China	UC	Moderate-severe	Naso-jejunal	Healthy donor	200–250 mL of fecal suspension	3 doses at 1, 3 and 5 days	-	-	12
Chen 2020 [43]	Prospective cohort	44	China	UC	Mild- moderate	Colonoscopy	Healthy donor	150–200 g stool/1000 mL saline	×3 in 1 week	-	-	12
Cold 2019 [44]	Prospective cohort	7	Denmark	UC	Active	Oral capsules	Healthy volunteers	12 g daily dose of 25 capsules	25 capsules/day for 50 days	-	-	24
Costello 2019 [35]	RCT	73	Australia	UC	Mild- moderate	Colonoscopy and enema	Healthy volunteers recruited by advertisement	50 g/200 mL saline colonoscopy, 25 g/100 mL saline enema	1× colonoscopy then 2× enemas over 1 week	-	-	8
Cui 2015 [45]	Prospective cohort	30	China	CD	Moderate-severe	Gastroscopy	Related or unrelated volunteer	60 mL/100 mL saline	×1	-	-	65
Dang 2020 [46]	Case series	12	China	UC	Moderate-severe	Colonoscopy	Healthy volunteers	15 mL bacterial pellet in 75 mL saline	multiple, exact frequency NR	-	-	52
Damman 2015 [47]	Prospective cohort	7	USA	UC	Mild- moderate	Colonoscopy	Chosen by patient	Diluted with 2–3 mL saline/g of stool	×1	-	-	12
Ishikawa 2017 [48]	Prospective cohort	36	Japan	UC	Mild-severe	Colonoscopy	Spouse or relative	150–250 g/350–500 mL saline	×1	Amoxicillin (1500 mg/day), Fosfomycin (3000 mg/day), metronidazole (750 mg/day)	2 weeks until 2 days before FMT	4
Jacob 2017 [49]	Prospective cohort	20	USA	UC	Active	Colonoscopy	Healthy donor	60 mL	×1	-	-	4
Kump 2017 [50]	Prospective cohort	27	Austria	UC	Mild-severe	Colonoscopy	Related or unrelated volunteer	50 g/200–500 mL saline	5×, 14 days apart	Vancomycin (250 mg qid), paromomycin (250 mg tid), nystatin (10 mL, 1 million IE qid)	10 days	13
Mizuno 2017 [51]	Prospective cohort	10	Japan	UC	Moderate-severe	Colonoscopy	Healthy relatives	50–300 g/50–100 mL saline	×1	-	-	12
Moayyedi 2015 [52]	RCT	70	Canada	UC	Mild-moderate	Enema	Healthy donors	50 g/300 mL water	×6; 0, 1, 2, 3, 4, 5, 6 weeks	-	-	7
Nishida 2017 [53]	Prospective cohort	41	Japan	UC	Mild-moderate	Colonoscopy	Healthy relatives	150–200 g/500 mL saline	×1	-	-	12
Okahara 2020 [54]	Prospective cohort	92	Japan	UC	Mild-severe	Colonoscopy	Spouses and relatives	350–500 mL filtered bacterial suspension infusion	×1	Amoxicillin (1500 mg/day), Fosfomycin (3000 mg/day), metronidazole (750 mg/day)	2 weeks prior to FMT	104
Paramsothy 2017 [55]	RCT	85	Australia	UC	Mild-moderate	Colonoscopy and enema	Healthy volunteers recruited by advertisement	37.5 g	×5/week for 8 weeks	-	-	8
Rossen 2015 [56]	RCT	50	Finland	UC	Mild- moderate	Nasoduodenal tube	Relatives, partner, or volunteer	120 g	×2; 3 weeks apart	-	-	12
Schierova 2020 [57]	Prospective cohort	16	Czech Republic	UC	NR	Enema	Healthy donors	50 g stool/150 mL saline	5×/week for 1 week then weekly × 6 weeks	-	-	12
Sokol 2020 [58]	RCT	17	France	CD	NR	Colonoscopy	Healthy donors	50–100 g/250–350 mL saline	×1	-	-	24
Sood 2019 [59]	Prospective cohort	41	India	UC	Mild- moderate	Colonoscopy	Two healthy unrelated volunteers	NR	×7; 0, 2, 6, 10, 14, 18, 22 weeks	-	-	22
Sood 2020 [60]	Prospective cohort	140	India	UC	Moderate-severe	Colonoscopy	Healthy donors	80 g stool/ 200 mL saline	×7; 0, 2, 6, 10, 14, 18, 22 weeks	-	-	30
Uygun 2017 [61]	Prospective cohort	30	Turkey	UC	Moderate-severe	Colonoscopy	Relatives, partner, or volunteer	120–150 g	×1	-	-	12
Vaughn 2016 [62]	Prospective cohort	19	USA	CD	Active	Colonoscopy	Healthy unrelated volunteers	50 g/250 mL saline	×1	-	-	4
Vermeire 2016 [63]	Prospective cohort	14	Belgium	UC+ CD	Refractory	Naso-jejunal or rectal tube	Family, friend, or partner	200 g/400 mL saline	×2; 2 consecutive days	-	-	8
Wang 2020 [64]	Prospective cohort	16	China	UC	Moderate- severe	Colonoscopy	Healthy donor	100 g stool/ 500 mL saline	×3; 2–3 month intervals	-	-	>24
Wei 2015 [34]	Prospective cohort	14	China	UC+ CD	NR	Colonoscopy or naso-jejunal tube	Healthy unrelated donor	60 g/350 mL saline	×1	Vancomycin (500 mg)	Twice a day for 3 days before FMT	4
Yang 2019 [65]	RCT	27	China	CD	Mild- moderate	Gastroscopy or colonoscopy	Healthy donors	200 g in saline	×2; 1 week apart	-	-	2
Zhang 2016 [66]	Prospective cohort	19	China	UC	Moderate-severe	Gastroscopy	NR	NR	×1	-	-	13

IBD—Inflammatory Bowel Disease; FMT—Fecal Microbiota Transplantation; UC—Ulcerative Colitis; CD—Crohn’s Disease; NR—Not recorded; RCT—Double-blinded, randomized controlled trial.

**Table 2:** Baseline characteristics of patients for included studies.

Study	Disease	Intervention Arm	Patients (n)	Age	Sex (% male)	Disease Duration (Years)	Ongoing Systemic Corticosteroids (%)	Total Mayo Score	CDAI
Borody 2003	UC	**Antibiotic pre-treatment and repeated FMT**	6	35.8 (11.0)	50.0	11.7 (5.8)	NR	NR	-
Chen 2020	UC	**Repeated FMT**	9	47.9 (10.6)	77.8	5.3 (5.1)	33.3	5.9 (2.0)	-
Chen 2020	UC	**Repeated FMT**	44	44.4 (15.5)	57	4.6 (2.1)	25.0	5.9 (2.0)	-
Cold 2019	UC	**Repeated FMT**	7	38.3 (5.8)	71.4	10.8 (3.8)	NR	NR	-
Costello 2019	UC	**Repeated donor FMT**	38	38.5 (6)	53.0	4.9 (4.8)	21.0	7.2 (1.7)	-
		**Repeated autologous FMT**	35	35.0 (5.25)	57.0	5.8 (2.2)	31.0	7.4 (1.9)	-
Cui 2015	CD	**Single FMT**	30	38.0 (13.8)	64.5	7.4 (5.3)	56.7	NR	NR
Damman 2015	UC	**Single FMT**	8	41.1 (15.5)	25.0	16.6 (13.1)	NR	NR	-
Dang 2020	UC	**Repeated FMT**	12	51 (14.0)	66.0	NR	41.7	NR	-
Ishikawa 2017	UC	**Antibiotic pre-treatment and single FMT**	17	40.4 (14.2)	76.5	7.8 (8.4)	29.4	7.5 (1.9)	-
		**Antibiotic pre-treatment only**	19	44.8 (14.9)	63.2	7.0 (8.0)	47.4	8.2 (2.2)	-
Jacob 2017	UC	**Single FMT**	20	38.4 (12.6)	60.0	NR	30.0	8.1 (2.4)	-
Kump 2017	UC	**Antibiotic pre-treatment and repeated FMT**	17	44.0 (18.0)	82.0	8.0 (8.0)	59.0	8.9 (1.6)	-
		**Antibiotic pre-treatment only**	10	36.0 (13.0)	30.0	7.0 (6.0)	30.0	8.1 (3.1)	-
Mizuno 2017	UC	**Single FMT**	10	31.8 (7.8)	70.0	6.25 (3.5)	NR	6.1 (1.0)	-
Moayyedi 2015	UC	**Repeated FMT**	38	42.2 (15.0)	47.0	7.9 (5.6)	39	8.2 (2.6)	-
		**Placebo**	37	35.8 (12.1)	70.0	7.0 (6.8)	35	7.9 (2.3)	-
Nishida 2017	UC	**Single FMT**	41	39.6 (16.9)	68.3	7.6 (8.6)	26.8	5.6 (2.4)	-
Okahara 2020	UC	**Antibiotic pre-treatment Single FMT**	55	40.1 (13.3)	69.1	8.6 (7.4)	43.2	6.3 (4.1)	-
Paramsothy 2017	UC	**Repeated FMT**	41	35.6 (5.3)	54.0	5.8 (1.4)	22.0	8 (0.8)	-
		**Placebo**	40	35.4 (4.5)	63.0	5.8 (1.4)	28.0	8 (0.8)	-
Rossen 2015	UC	**Single donor FMT**	23	42.3 (5.8)	47.8	7 (NR)	21.7	NR	-
		**Single autologous FMT**	25	41 (4.5)	44.0	9 (NR)	20.0	NR	-
Schierova 2020	UC	**Repeated FMT**	8	41.3 (10.1)	50.0	NR	0	5.8 (1.7)	-
		**Medical therapy**	8	44.3 (10.4)	50.0	NR	25.0	6.0 (1.5)	-
Sokol 2020	CD	**Single FMT**	8	31.8 (6.8)	62.5	8.5 (8.1)	100	NR	89 (30.5)
		**Placebo**	9	38.3 (6.0)	44.4	11.3 (2.0)	100	NR	61.5 (20.1)
Sood 2019	UC	**Repeated FMT**	41	36.5 (10.7)	58.5	4.6 (4.2)	100	8.8 (2.6)	-
Sood 2020	UC	**Repeated FMT**	93	35 (11)	62.4	5.2 (4.6)	78.5	8.1 (2.0)	-
Uygun 2017	UC	**Single FMT**	30	34.6 (10.3)	46.7	5.3 (3.3)	NR	11.1 (1.1)	-
Vaughn 2016	CD	**Single FMT**	19	36 (12.3)	63.0	12.5 (10.6)	42.0	NR	NR
Vermeire 2016	UC and CD	**Repeated FMT**	14	38.6 (8.2)	50.0	10.2 (7.5)	21.4	8.4 (0.6)	290 (29)
Wang 2020	UC	**Repeated FMT**	16	39.5 (4)	62.5	7.5 (5.8)	NR	9.9 (2.2)	-
Wei 2015	UC and CD	**Antibiotic pre-treatment and single FMT**	14	43.5 (16.4)	42.9	4.1 (3.2)	7.1	5.8 (1.9)	345 (77.8)
Yang 2019	CD	**Repeated FMT**	30	72.2 (10.8))	57.9	1.3 (0.4)	NR	NR	283 (131)
Zhang 2016	UC	**Single FMT**	19	39.2 (14.1)	36.8	8.0 (5.8)	NR	10.5 (1.7)	-

Values are presented as mean +/− SD; UC—ulcerative colitis; CD—Crohn’s disease; NR—Not Recorded; CDAI—Crohn’s Disease Activity Index.

**Table 3:** Response and remission rates for included studies.

Study	Intervention Arm	Follow-Up at Response/Remission (Weeks)	Patients (*n*)	Response (%)	Remission (%)
Borody 2003	Antibiotic pre-treatment and repeated FMT	676	6	6 (100%)	6 (100%)
Chen 2020	Repeated FMT	2 weeks for response 12 weeks for remission	9	7 (77.8%)	5 (55.6%)
Chen 2020	Repeated FMT	12	44	34 (77.3%)	30 (68.2%)
Cold 2019	Repeated FMT	24	7	7 (100%)	4 (57.1%)
Costello 2019	Repeated donor FMT	8	38	21 (55%)	18 (47%)
	Repeated autologous FMT	8	35	8 (23%)	6 (17%)
Cui 2015	Single FMT	12–72	15	8 (53.3%)	4 (26.7%)
Dang 2020	Repeated FMT	52	12	11 (91.7%)	5 (41.7%)
Damman 2015	Single FMT	4	7	1 (14.3%)	1 (14.3%)
Ishikawa 2017	Antibiotic pre-treatment and single FMT	4	17	14 (82.3%)	9 (52.9%)
	Antibiotic pre-treatment only	4	19	13 (68.4%)	3 (15.8%)
Jacob 2017	Single FMT	4	20	7 (35%)	3 (15%)
Kump 2017	Antibiotic pre-treatment and repeated FMT	13	17	10 (59%)	4 (24%)
	Antibiotic pre-treatment only	13	10	1 (10%)	0 (0%)
Mizuno 2017	Single FMT	12	10	1 (10%)	0 (0%)
Moayyedi 2015	Repeated FMT	7	38	15 (39%)	9 (24%)
	Placebo	7	37	9 (24%)	2 (5%)
Nishida 2017	Single FMT	8	41	11 (26.8%)	0 (0%)
Okahara 2020	Single FMT	4	55	31 (56.3%)	19 (34.5%)
Paramsothy 2017	Repeated FMT	8	41	22 (54%)	18 (44%)
	Placebo	8	40	9 (23%)	8 (20%)
Rossen 2015	Repeated donor FMT	12	23	11 (47.8%)	7 (30.4%)
	Repeated autologous FMT	12	25	13 (52.0%)	8 (32.0%)
Schierova 2020	Repeated FMT	12	8	5 (62.5%)	1 (12.5%)
Sokol 2020	Single FMT	24	8	NR	4 (50%)
Sood 2019	Repeated FMT	22	41	31 (75.6%)	19 (46.3%)
Sood 2020	Repeated FMT	30	93	NR	57 (61.3%)
Uygun 2017	Single FMT	12	30	21 (70%)	13 (43.3%)
Vaughn 2016	Single FMT	4	19	11 (58%)	10 (53%)
Vermeire 2016	Repeated FMT	6 weeks for response 8 weeks for remission	14	4 (50%)	2 (14.3%)
Wang 2020	Repeated FMT	>6 mo	16	14 (87.5%)	0 (0%)
Wei 2015	Antibiotic pre-treatment and single FMT	4	14	14 (100%)	14 (100%)
Yang 2019	Repeated FMT	2	27	21 (77.8%)	18 (66.7%)
Zhang 2016	Single FMT	13	19	11 (57.9%)	2 (10.5%)

**Table 4:** Effect of fecal microbial transplant therapy on microbiota composition.

Study	Methods	Donor Microbiota Differences vs. Recipient	Recipient Microbiota Changes Following FMT	Recipient Microbiota Changes Associated with Response/Remission
Borody 2003	NR	NR	NR	NR
Chen 2020	NR	NR	NR	NR
Chen 2020	16 s rRNA	↑ α—diversity (Shannon, Chao1)	↑ α—diversity (Shannon, Chao1) ↑ *F. Prausnitzii*	NR
Cold 2019	16 s rRNA	NR	No change in α—diversity (Shannon, Simpson)	NR
Costello 2019	16 s rRNA	NR	↑ α—diversity (operational taxonomic units—OTUs) ↑ *Peptococcus niger*, ↑ *Faecalicoccus pleomorphus*, ↑ *Olsenella* sp., ↑ *Acidaminococcus intestini*, ↑ *Prevotella copri*, ↑ *Clostridium methylpentosum*, ↑ *Allistipes indistinctus*, ↑ *Odoribacter splanchnicus* ↓ *Anaerostipescaccae*, ↓ *Clostridium aldenense*	NR
Cui 2015	NR	NR	NR	NR
Damman 2015	Metagenomic Shotgun Sequencing	NR	No significant difference in α diversity (Shannon) ↑ *Actinobacteria*, ↑ *Bacteroidetes* *(Prevotella copri)*	NR
Dang 2020	NR	NR	NR	NR
Ishikawa 2017	16 s rDNA	NR	↑ *Bacteroidetes*	NR
Jacob 2017	16 s rRNA	NR	↑ α—diversity (OTUs, Shannon) Change in β—diversity (Bray-Curtis) towards donor	NR
Kump 2017	16 s rRNA	↑ unclassified *Ruminococcus* sp., ↑ *Akkermansia muciniphila*	No change in α—diversity (Chao1) Change in β—diversity (Bray-Curtis) towards donor	↑ *Akkermansia*, ↓ *Dialister* sp. Change in β—diversity (Bray-Curtis) towards donor in responders
Mizuno 2017	16 s rRNA	NR	No significant difference in diversity or composition	NR
Moayyedi 2015	16 s rRNA	↑ *Lachnospiraceae*, ↑ *Ruminococcus*	Change in β—diversity (Bray-Curtis) towards donor	Change in β—diversity (Bray-Curtis) towards donor
Nishida 2017	16 s rRNA	↑ *Bifidobacterium*	No significant difference in α—diversity (Shannon) or β—diversity (Bray-Curtis) between responders and non-responders	NR
Okahara 2020	HSP60 Bacteroidetes Sequencing	NR	Increase in similarity of *Bacteroidetes* species to donor	*↑ Bacteroides uniformis*, *↑ Parabacteroides distasonis*, *↑ Bacteroides dorei*
Paramsothy 2017	16 s rRNA shotgun sequencing	NR	↑ α—diversity (OTUs, Shannon) Shift towards donor at OTU level ↑ *Prevotella* spp., *↓* *Bacteroides* spp.	*↑Barnesiella* spp., *↑ Parabacteroides* spp., *↑* *Clostridium cluster IV*, *↑* *Ruminococcus* spp.
Rossen 2015	16 s rRNA	NR	↑ α—diversity (OTUs, Shannon) ↑ *Clostridium clusters IV*, *XIVa, XVIII* ↓ *Bacteroidetes*	NR
Schierova 2020	16 sRNA	NR	No difference in α—diversity (Shannon, Chao1, Faith’s phylogenetic diversity) or β—diversity	*↑ Lachnospiraceae*, *↑ Ruminococcaeae*, *↑ Clostridaceae*, *↑ Bifidobacteriaceae*, *↑ Coriobacteriaceace* *↑Faecalibacterium* *↑ Blautia*, *↑ Coriobacteria*, *↑ Collinsella*, *↑ Slackia*, *↑ Bifidobacterium*
Sokol 2020	16 s rRNA	NR	Transient ↑ α—diversity (Shannon, Chao1) Trend towards change in β—diversity (Bray-Curtis, Sorensen similarity index) between donor/recipient correlated	Sorensen index similarity showing improved engraftment; *↑ Ruminococcaecea*, *↑ Coprococcus*, *↑ Desulfovibrio*
Sood 2019	NR	NR	NR	NR
Sood 2020	NR	NR	NR	NR
Uygun 2017	NR	NR	NR	NR
Vaughn 2016	Whole-genome shotgun sequencing	NR	↑ α—diversity (Shannon) ↑ *Bacteroides cellulosilyticus*, *↑ Bilophila* unclassified, *↑* *Desulfovibrio piger*, *↑* *Bilophila wadsorthia*, *↑* *Clostridium leptum*, *↑* *Odoribacter splanchnicus*, *↑* *Bacteroides dorei*, *↑* *Parasutterella excrementihominis*, *↑* *Lachnospiraceae bacterium 7 1 58FAA*, *↑* *Eubacterium ventriosum*, *↑ Burkholderiales bacterium 1 1 47*, *↑* *Dorea longicatena*, *↑* *Alistipes finegoldii* ↓ *Coprobacillus* unclassified, *↓* *Bacteroides massiliensis*, *↓* *Ruminococcus lactaris*, *↓* *Veillonella dispar*, *↓* *Lachnospiraceae bacterium 5 1 57FAA*, *↓* *Bifidobacterium adolescentis*, *↓* *Bacteroides vulgatus*, *↓* *Bacteroides ovatus*, *↓ Streptococcus parasanguinis*, *↓* *Streptococcus salivarius*, *↓ Clostridium scindens*	Change in β—diversity (Bray-Curtis) towards donor in responders
Vermeire 2016	16 s DNA	↑ α—diversity (OTUs)	↑ α—diversity (OTUs), ↑ *Roseburia*, *Oscillibacter*, ↑ unclassified *Lachnospiraceae*, ↑ unclassified *Ruminococcaceae*	NR
Wang 2020	NR	NR	NR	NR
Wei 2015	NR	NR	NR	NR
Yang 2019	16 s RNA	NR	↑ α—diversity (OTUs, Shannon)	NR
Zhang 2016	NR	NR	NR	NR

NR—Not recorded.

**Table 5:** Adverse events and interventions reported for included studies.

Study	FMT or Antibiotic Treatment Delivery and Frequency	Patients (n)	Adverse Events Per Patient	Action
Borody 2003	Daily enema for 5 days	6	NR	NR
Chen 2020	Naso-jejunal 3 doses at 1, 3 and 5 days	9	Mild bloating (*n* = 3) Treatment failure (*n* = 1)	Colectomy (*n* = 1)
Chen 2020	Colonoscopy ×3 in 1 week	44	NR	NR
Cold 2019	25 oral capsules per day for 50 days	7	No adverse events	No adverse events
Costello 2019	Single donor FMT (colonoscopy and 2 enemas over a week)	38	After 8 weeks: Worsening colitis (*n* = 1) *C. difficile* infection (*n* = 1) Pneumonia (*n* = 1) New anemia (*n* = 1) Mild elevation of alkaline phosphatase (*n* = 2) and alanine aminotransferase (*n* = 1)	Colectomy (*n* = 1)
	Single autologous FMT (colonoscopy and 2 enemas over a week)	35	After 8 weeks: Worsening colitis (*n* = 2) New anemia (*n* = 2) Mild elevation of alanine aminotransferase (*n* = 3)	NR
		61	After 12 months: Worsening colitis (*n* = 13) Infections (*n* = 8) New psoriatic arthritis (*n* = 2) Entero-pathic arthritis (*n* = 1) Crohn’s disease (*n* = 1) Allergy to infliximab (*n* = 1) Weight gain (*n* = 13) Weight loss (*n* = 8)	Colectomy (*n* = 9)
Cui 2015	Single gastroscopy	30	Fever (*n* = 2)—1–6 h after FMT Increased diarrhea (*n* = 7)—1–6 h after FMT	NR
Damman 2015	Single colonoscopy	7	Abdominal cramping, increase in stool output (NR)—immediately after FMT Abdominal pain (*n* = 1)—after 5 days	None
Ishikawa 2017	Single colonoscopy	21	Transient borborygmus (*n* = 10)—during or soon after FMT	Resolved after end of treatment (*n* = 10)
	Antibiotic pre-treatment only	20	Nausea and watery diarrhea—after antibiotic treatment (*n* = 8)	Discontinued antibiotic treatment (*n* = 3)
Jacob 2017	Single colonoscopy	20	Fever (*n* = 1) Chills (*n* = 1) Fatigue/malaise (*n* = 4) Abdominal pain (*n* = 3) Anorexia (*n* = 1) Diarrhea (*n* = 2) Constipation (*n* = 1) Transient febrile response (*n* = 1) Increase in Mayo score (*n* = 2)—at week 4	Conservative care Anti-TNF alpha blockade therapy or colectomy
Kump 2017	Colonoscopy (5 times, 14 days apart)	17	Worsening colitis (*n* = 1)—after day 3	Required additional therapy (*n* = 1)
	Antibiotic pre-treatment only	10	*C. difficile* infection (*n* = 3)—after 14 days Antibiotic-associated diarrhea (*n* = 1) Worsening colitis (*n* = 1)	Required additional therapy (*n* = 5)
Mizuno 2017	Single colonoscopy	10	Worsening colitis (*n* = 6)	
Moayyedi 2015	Enema (once per week for 6 weeks)	38	Patchy inflammation and rectal abscess (*n* = 2) Abdominal discomfort (*n* = 1) *C. difficile* infection (*n* = 1)—after end of study	Antibiotic therapy (*n* = 2)
	Placebo	37	Worsening colitis (*n* = 1) Patchy inflammation and rectal abscess (*n* = 1)	Colectomy (*n* = 1) Antibiotic therapy (*n* = 1)
Nishida 2017	Single colonoscopy	41	No adverse events	
Okahara 2020	Single colonoscopy	55	Nausea (*n* = 20)	None
Paramsothy 2017	Colonoscopy and enema (×5 per week for 8 weeks)	41	Infection-related adverse event (*n* = 10) Serious adverse event (*n* = 2) Abdominal pain (*n* = 12) Colitis (*n* = 10) Flatulence (*n* = 10) Bloating (*n* = 8) Upper respiratory tract infection (*n* = 7) Headache (*n* = 4) Dizziness (*n* = 3) Fever (*n* = 3) Rash (*n* = 3)	Colectomy (*n* = 1), intravenous corticosteroid therapy (*n* = 1)
	Placebo	40	Infection-related adverse event (*n* = 14) Serious adverse event (*n* = 1) Abdominal pain (*n* = 11) Colitis (*n* = 9) Flatulence (*n* = 8) Bloating (*n* = 11) Upper respiratory tract infection (*n* = 6) Headache (*n* = 2) Dizziness (*n* = 3) Fever (*n* = 2)	Hospitalization (*n* = 1)
Rossen 2015	Donor FMT by nasoduodenal tube (twice, 3 weeks apart)	23	Discomfort with tube placement (*n* = 1) Fever (*n* = 2) Nausea (*n* = 2) Diarhea (*n* = 5) Headache (*n* = 1) Vomited fecal infusion (*n* = 2) Vomiting (*n* = 1) Abdominal pain (*n* = 1) Transient borborygmus (*n* = 4) Mild constipation (*n* = 1)	
	Autologous FMT by nasoduodenal tube (twice, 3 weeks apart)	25	Discomfort with tube placement (*n* = 1) Nausea (*n* = 1) Malaise (*n* = 1) Diarrhea (*n* = 1) Headache (*n* = 1) Abdominal cramps (*n* = 6) Abdominal pain (*n* = 4) Transient borborygmus (*n* = 8) Dizziness (*n* = 1) Cytomegalovirus infection (*n* = 1)—7 weeks after the first FMT; unrelated to treatment	Ganciclovir (*n* = 1)
		50	Severe small bowel Crohn’s disease (*n* = 1) Abdominal pain (*n* = 1)—after 11 weeks Cervix carcinoma (*n* = 1)—after 6 weeks; unrelated to treatment	Antibiotics (*n* = 1)
Schierova 2020	Enema 5× for first week then weekly × 6 weeks	8	No adverse events	None
Sokol 2020	Single colonoscopy	8	Gastroenteritis (*n* = 2) Transient asthenia (*n* = 1) Cutaneous abscess (*n* = 1)	Self-limiting
Sood 2019	Colonoscopy at 0, 2, 6, 10, 14, 18, 22 weeks	41	After FMT, at 0 weeks: Abdominal discomfort (*n* = 26) Abdominal distension (*n* = 14) Fever (*n* = 4) Worsening diarrhea (*n* = 4) Flatulence (*n* = 2) Fatigue (*n* = 2)	Symptoms were self-limiting Oral rehydration solution (*n* = 4)
Sood 2020	Colonoscopy at 0, 2, 6, 10, 14, 18, 22 weeks	93	Abdominal discomfort (*n* = 28) Flatulence (*n* = 12) Borborygmi (*n* = 10) Low grade fever (*n* = 8) Diarrhea (*n* = 7)	Self-limiting
Uygun 2017	Single colonoscopy	30	Nausea, vomiting, abdominal pain, diarrhea (*n* = 7)	NR
Vaughn 2016	Single colonoscopy	19	Hives (*n* = 1)	Oral steroids (*n* = 1)
Vermeire 2016	Naso-jejunal or rectal tube (twice one day, then the following day)	14	High fever (*n* = 4)—few hours after FMT Vomited and pneumonia (*n* = 1)—after FMT	Paracetamol (*n* = 4) Broad-spectrum antibiotics (*n* = 1)
Wang 2020	Colonoscopy ×3; 2–3 month intervals	16	None	None
Wei 2015	Single colonoscopy or naso-jejunal tube	14	Intolerance with FMT (*n* = 1) Moderate fever (*n* = 2)—after FMT	Self-limiting
Yang 2019	Gastroscopy or colonoscopy (twice, one week apart)	31	Nausea (*n* = 1) Reflux (*n* = 4) Belching (*n* = 2) Diarrhea (*n* = 10) Constipation (*n* = 1) Fever (*n* = 2) Aggravation of abdominal pain (*n* = 5) Abdominal distension (*n* = 3)	NR
Zhang 2016	Single endoscopy	19	Transient increased diarrhea (*n* = 7) Mild skin pruritus (*n* = 1) Borborygmus (*n* = 2)	-

NR—Not recorded.

## Supplementary Materials

The following are available online at https://www.mdpi.com/2077-0383/10/5/959/s1, Table S1: Full-text search strategy of included databases; Table S2: Newcastle-Ottawa scale for assessing risk of bias for included cohort studies; Table S3: Cochrane risk of bias assessment for included randomized trials.

## References

[1] Loftus E.V. (2004). Clinical epidemiology of inflammatory bowel disease: Incidence, prevalence, and environmental influences. Gastroenterology.

[2] Nikolaus S., Schreiber S. (2007). Diagnostics of Inflammatory Bowel Disease. Gastroenterology.

[3] Molodecky N.A., Soon I.S., Rabi D.M., Ghali W.A., Ferris M., Chernoff G., Benchimol E.I., Panaccione R., Ghosh S., Barkema H.W. (2012). Increasing Incidence and Prevalence of the Inflammatory Bowel Diseases With Time, Based on Systematic Review. Gastroenterology.

[4] Palmela C., Chevarin C., Xu Z., Torres J., Sevrin G., Hirten R., Barnich N., Ng S.C., Colombel J.F. (2018). Adherent-invasive Escherichia coli in inflammatory bowel disease. Gut.

[5] Kim D.H., Cheon J.H. (2017). Pathogenesis of Inflammatory Bowel Disease and Recent Advances in Biologic Therapies. Immune Netw..

[6] Wu H., Tremaroli V., Backhed F. (2015). Linking Microbiota to Human Diseases: A Systems Biology Perspective. Trends Endocrinol. Metab..

[7] Wu G.D., Lewis J.D. (2013). Analysis of the human gut microbiome and association with disease. Clin. Gastroenterol. Hepatol..

[8] McIlroy J., Ianiro G., Mukhopadhya I., Hansen R., Hold G.L. (2018). The gut microbiome in inflammatory bowel disease-avenues for microbial management. Aliment. Pharmacol. Ther..

[9] Philpott J., Ashburn J., Shen B. (2017). Efficacy of vedolizumab in patients with antibiotic and anti-tumor necrosis alpha refractory pouchitis. Inflamm. Bowel Dis..

[10] Wilhelm S.M., Love B.L. (2017). Management of patients with inflammatory bowel disease: Current and future treatments. Clin. Pharm..

[11] Furfaro F., Fiorino G., Allocca M., Gilardi D., Danese S. (2016). Emerging therapeutic targets and strategies in Crohn’s disease. Expert Rev. Gastroenterol. Hepatol..

[12] Abu-Sbeih H., Wang Y. (2019). Management Considerations for Immune Checkpoint Inhibitor-Induced Enterocolitis Based on Management of Inflammatory Bowel Disease. Inflamm. Bowel Dis..

[13] Torres J., Danese S., Colombel J. (2013). New therapeutic avenues in ulcerative colitis: Thinking out of the box. Gut.

[14] Chande N., Costello S.P., Limketkai B.N., Parker C.E., Nguyen T.M., Macdonald J.K., Feagan B.G. (2019). Alternative and Complementary Approaches for the Treatment of Inflammatory Bowel Disease: Evidence From Cochrane Reviews. Inflamm. Bowel Dis..

[15] Maharshak N., Cohen N.A., Reshef L., Tulchinsky H., Gophna U., Dotan I. (2016). Low enteric microbial diversity in patients with ulcerative colitis after pouch surgery having a mature normal ileal pouch may be predictive of pouchitis. J. Crohn’s Colitis.

[16] Ishikawa D., Osada T., Sasaki T., Kuwahara-Arai K., Haga K., Shibuya T., Kodani T., Hiramatsu K., Watanabe S. (2015). Alterations of intestinal microbiota in ulcerative colitis patients treated with sequential antibiotic combination and faecal microbiota transplantation. J. Crohn’s Colitis.

[17] Zuo T., Ng S.C. (2018). The Gut Microbiota in the Pathogenesis and Therapeutics of Inflammatory Bowel Disease. Front. Microbiol..

[18] Pigneur B., Sokol H. (2016). Fecal microbiota transplantation in inflammatory bowel disease: The quest for the holy grail. Mucosal Immunol..

[19] Gallo A., Passaro G., Gasbarrini A., Landolfi R., Montalto M. (2016). Modulation of microbiota as treatment for intestinal inflammatory disorders: An uptodate. World J. Gastroenterol..

[20] Zeng W., Shen J., Bo T., Peng L., Xu H., Nasser M.I., Zhuang Q., Zhao M. (2019). Cutting Edge: Probiotics and Fecal Microbiota Transplantation in Immunomodulation. J. Immunol. Res..

[21] Weingarden A.R., Vaughn B.P. (2017). Intestinal microbiota, fecal microbiota transplantation, and inflammatory bowel disease. Gut Microbes.

[22] Paramsothy S., Paramsothy R., Kamm M.A., Kaakoush N.O., Mitchell H.M., Rubin D.T., Castano-Rodriguez N. (2017). Faecal microbiota transplantation for inflammatory bowel disease: A systematic review and meta-analysis. Gastroenterology.

[23] Khoruts A., Rank K.M., Newman K.M., Viskocil K., Vaughn B.P., Hamilton M.J., Sadowsky M.J. (2016). Inflammatory Bowel Disease Affects the Outcome of Fecal Microbiota Transplantation for Recurrent Clostridium difficile Infection. Clin. Gastroenterol. Hepatol..

[24] Khanna S., Vazquez-Baeza Y., Gonazlez A., Weiss S., Schmidt B., Muniz-Pedrogo D.A., Rainey J.F., Kammer P., Nelson H., Sadowsky M. (2017). Changes in microbial ecology after fecal microbiota transplantation for recurrent C-difficile infection affected by underlying inflammatory bowel disease. Microbiome.

[25] Langdon A., Crook N., Dantas G. (2016). The effects of antibiotics on the microbiome throughout development and alternative approaches for therapeutic modulation. Genome Med..

[26] Khoruts A., Sadowsky M.J. (2016). Understanding the mechanisms of faecal microbiota transplantation. Nat. Rev. Gastroenterol. Hepatol..

[27] Ishikawa D., Takahashi M., Ito S., Okahara K., Haga K., Kamei M., Nomura O., Shibuya T., Osada T., Nagahara A. (2018). Eradication of dysbiotic indigenous species by multiple antibiotic pre-treatment contribute to effective faecal microbiota transplantation. J. Crohn’s Colitis.

[28] Ishikawa D., Sasaki T., Takahashi M., Okahara K., Ito S., Haga K., Shibuya T., Osada T., Nagahara A. (2019). Combination therapy of fresh fecal microbial transplantation and triple-antibiotic therapy for ulcerative colitis. Am. J. Gastroenterol..

[29] Shimizu H., Ohnishi E., Arai K., Takeuchi I., Kamura H., Hata K. (2019). Outcome of the repetitive fecal microbiota transplantation using fecal solution prepared under the anaerobic condition following the antibiotic pretreatment in eight children with ulcerative colitis. Inflamm. Bowel Dis..

[30] Blesl A., Rainer F., Wurm P., Durdevic M., Petritsch W., Wenzl H., Baumann-Durchschein F., Posch A., Streit A., Gorkiewicz G. (2019). Predictors of non-response to repeated faecal microbiota transplantation in patients with therapy refractory ulcerative colitis. J. Crohn’s Colitis.

[31] Borody T.J., Warren E.F., Leis S., Surace R., Ashman O. (2003). Treatment of ulcerative colitis using fecal bacteriotherapy. J. Clin. Gastroenterol..

[32] Ishikawa D., Okahara K., Ito S., Takahashi M., Haga K., Nomura K., Shibuya T., Nagahara A. (2019). Efficacy of combination of fresh fecal microbiota transplantation and triple-antibiotic therapy for ulcerative colitis. Inflamm. Bowel Dis..

[33] Ishikawa D., Takahashi M., Okahara K., Ito S., Haga K., Shibuya T., Osada T., Nagahara A. (2019). Efficacy of combination therapy of fresh faecal microbiota transplantation and triple-antibiotic therapy for ulcerative colitis. J. Crohn’s Colitis.

[34] Wei Y., Zhu W., Gong J., Guo D., Gu L., Li N., Li J. (2015). Fecal Microbiota Transplantation Improves the Quality of Life in Patients with Inflammatory Bowel Disease. Gastroenterol. Res. Pract..

[35] Costello S.P., Hughes P.A., Waters O., Bryant R.V., Vincent A.D., Blatchford P., Katsikeros R., Makanyanga J., Campaniello M.A., Mavrangelos C. (2019). Effect of Fecal Microbiota Transplantation on 8-Week Remission in Patients With Ulcerative Colitis: A Randomized Clinical Trial. JAMA.

[36] Sood A., Mahajan R., Singh A., Midha V., Mehta V., Narang V., Singh T., Singh Pannu A. (2019). Role of Faecal Microbiota Transplantation for Maintenance of Remission in Patients With Ulcerative Colitis: A Pilot Study. J. Crohn’s. Colitis.

[37] Stang A. (2010). Critical evaluation of the Newcastle-Ottawa scale for the assessment of the quality of nonrandomized studies in meta-analyses. Eur. J. Epidemiol..

[38] Higgins J.P.T. (2011). Cochrane Handbook for Systematic Reviews of Interventions Version 5.1.0.

[39] Hozo S.P., Djulbegovic B., Hozo I. (2005). Estimating the mean and variance from the median, range, and the size of a sample. BMC Med. Res. Methodol..

[40] Higgins J.P.T., Thomas J., Chandler J., Cumpston M., Li T., Page M.J., Welch V.A. (2019). Chapter 10: Analysing Data and Undertaking Meta-Analyses.

[41] Higgins J.P.T., Thompson S.G., Deeks J.J., Altman D.G. (2003). Measuring inconsistency in meta-analyses. BMJ.

[42] Chen M., Liu X.L., Zhang Y.J., Nie Y.Z., Wu K.C., Shi Y.Q. (2020). Efficacy and safety of fecal microbiota transplantation by washed preparation in patients with moderate to severely active ulcerative colitis. J. Dig. Dis..

[43] Chen H.T., Huang H.L., Xu H.M., Luo Q.L., He J., Li Y.Q., Zhou Y.L., Nie Y.Q., Zhou Y.J. (2020). Fecal microbiota transplantation ameliorates active ulcerative colitis. Exp. Ther. Med..

[44] Cold F., Browne P.D., Gunther S., Halkjaer S.I., Petersen A.M., Al-Gibouri Z., Hansen L.H., Christensen A.H. (2019). Multidonor FMT capsules improve symptoms and decrease fecal calprotectin in ulcerative colitis patients while treated—An open-label pilot study. Scand. J. Gastroenterol..

[45] Cui B., Feng Q., Wang H., Wang M., Peng Z., Li P., Huang G., Liu Z., Wu P., Fan Z. (2015). Fecal microbiota transplantation through mid-gut for refractory Crohn’s disease: Safety, feasibility, and efficacy trial results. J. Gastroenterol. Hepatol..

[46] Dang X.F., Wang Q.X., Yin Z., Sun L., Yang W.H. (2020). Recurrence of moderate to severe ulcerative colitis after fecal microbiota transplantation treatment and the efficacy of re-FMT: A case series. BMC Gastroenterol..

[47] Damman C.J., Brittnacher M.J., Westerhoff M., Hayden H.S., Radey M., Hager K.R., Marquis S.R., Miller S.I., Zisman T.L. (2015). Low Level Engraftment and Improvement following a Single Colonoscopic Administration of Fecal Microbiota to Patients with Ulcerative Colitis. PLoS ONE.

[48] Ishikawa D., Sasaki T., Osada T., Kuwahara-Arai K., Haga K., Shibuya T., Hiramatsu K., Watanabe S. (2017). Changes in Intestinal Microbiota Following Combination Therapy with Fecal Microbial Transplantation and Antibiotics for Ulcerative Colitis. Inflamm. Bowel Dis..

[49] Jacob V., Crawford C., Cohen-Mekelburg S., Viladomiu M., Putzel G.G., Schneider Y., Chabouni F., O’Neil S., Bosworth B., Woo V. (2017). Single Delivery of High-Diversity Fecal Microbiota Preparation by Colonoscopy Is Safe and Effective in Increasing Microbial Diversity in Active Ulcerative Colitis. Inflamm. Bowel Dis..

[50] Kump P., Wurm P., Grochenig H.P., Wenzl H., Petritsch W., Halwachs B., Wagner M., Stadlbauer V., Eherer A., Hoffmann K.M. (2018). The taxonomic composition of the donor intestinal microbiota is a major factor influencing the efficacy of faecal microbiota transplantation in therapy refractory ulcerative colitis. Aliment. Pharmacol. Ther..

[51] Mizuno S., Nanki K., Matsuoka K., Saigusa K., Ono K., Arai M., Sugimoto S., Kiyohara H., Nakashima M., Takeshita K. (2017). Single fecal microbiota transplantation failed to change intestinal microbiota and had limited effectiveness against ulcerative colitis in Japanese patients. Intest. Res..

[52] Moayyedi P., Surette M.G., Kim P.T., Libertucci J., Wolfe M., Onischi C., Armstrong D., Marshall J.K., Kassam Z., Reinisch W. (2015). Fecal Microbiota Transplantation Induces Remission in Patients With Active Ulcerative Colitis in a Randomized Controlled Trial. Gastroenterology.

[53] Nishida A., Imaeda H., Ohno M., Inatomi O., Bamba S., Sugimoto M., Andoh A. (2017). Efficacy and safety of single fecal microbiota transplantation for Japanese patients with mild to moderately active ulcerative colitis. J. Gastroenterol..

[54] Okahara K., Ishikawa D., Nomura K., Ito S., Haga K., Takahashi M., Shibuya T., Osada T., Nagahara A. (2020). Matching between Donors and Ulcerative Colitis Patients Is Important for Long-Term Maintenance after Fecal Microbiota Transplantation. J. Clin. Med..

[55] Paramsothy S., Kamm M.A., Kaakoush N.O., Walsh A.J., van den Bogaerde J., Samuel D., Leong R.W.L., Connor S., Ng W., Paramsothy R. (2017). Multidonor intensive faecal microbiota transplantation for active ulcerative colitis: A randomised placebo-controlled trial. Lancet.

[56] Rossen N.G., MacDonald J.K., de Vries E.M., D’Haens G.R., de Vos W.M., Zoetendal E.G., Ponsioen C.Y. (2015). Fecal microbiota transplantation as novel therapy in gastroenterology: A systematic review. World J. Gastroenterol..

[57] Schierova D., Brezina J., Mrazek J., Fliegerova K.O., Kvasnova S., Bajer L., Drastich P. (2020). Gut Microbiome Changes in Patients with Active Left-Sided Ulcerative Colitis after Fecal Microbiome Transplantation and Topical 5-aminosalicylic Acid Therapy. Cells.

[58] Sokol H., Landman C., Seksik P., Berard L., Montil M., Nion-Larmurier I., Bourrier A., Le Gall G., Lalande V., De Rougemont A. (2020). Fecal microbiota transplantation to maintain remission in Crohn’s disease: A pilot randomized controlled study. Microbiome.

[59] Sood A., Mahajan R., Juyal G., Midha V., Grewal C.S., Mehta V., Singh A., Joshi M.C., Narang V., Kaur K. (2019). Efficacy of fecal microbiota therapy in steroid dependent ulcerative colitis: A real world intention-to-treat analysis. Intest. Res..

[60] Sood A., Singh A., Mahajan R., Midha V., Kaur K., Singh D., Bansal N., Dharni K. (2020). Clinical Predictors of response to Faecal Microbiota Transplantation in patients with active ulcerative colitis. J. Crohn’s. Colitis.

[61] Uygun A., Ozturk K., Demirci H., Oger C., Avci I.Y., Turker T., Gulsen M. (2017). Fecal microbiota transplantation is a rescue treatment modality for refractory ulcerative colitis. Medicine.

[62] Vaughn B.P., Vatanen T., Allegretti J.R., Bai A., Xavier R.J., Korzenik J., Gevers D., Ting A., Robson S.C., Moss A.C. (2016). Increased Intestinal Microbial Diversity Following Fecal Microbiota Transplant for Active Crohn’s Disease. Inflamm. Bowel Dis..

[63] Vermeire S., Joossens M., Verbeke K., Wang J., Machiels K., Sabino J., Ferrante M., Van Assche G., Rutgeerts P., Raes J. (2016). Donor Species Richness Determines Faecal Microbiota Transplantation Success in Inflammatory Bowel Disease. J. Crohn’s. Colitis.

[64] Wang Y., Ren R., Sun G., Peng L., Tian Y., Yang Y. (2020). Pilot study of cytokine changes evaluation after fecal microbiota transplantation in patients with ulcerative colitis. Int. Immunopharmacol..

[65] Yang Z., Bu C., Yuan W., Shen Z., Quan Y., Wu S., Zhu C. (2020). Fecal Microbiota Transplant via Endoscopic Delivering Through Small Intestine and Colon: No Difference for Crohn’s Disease. Dig. Dis. Sci..

[66] Zhang T., Cui B., Li P., Zhang F. (2016). Short-term surveillance of cytokines and CRP cannot predict efficacy of fecal microbiota transplantation for ulcerative colitis. Gastroenterology.

[67] Yu E.W., Gao L., Stastka P., Cheney M.C., Mahabamunuge J., Torres Soto M., Ford C.B., Bryant J.A., Henn M.R., Hohmann E.L. (2020). Fecal microbiota transplantation for the improvement of metabolism in obesity: The FMT-TRIM double-blind placebo-controlled pilot trial. PLOS Med..

[68] El-Salhy M., Mazzawi T. (2018). Fecal microbiota transplantation for managing irritable bowel syndrome. Expert Rev. Gastroenterol. Hepatol..

[69] Baunwall S.M., Lee M.M., Eriksen M.K., Mullish B.H., Marchesi J.R., Dahlerup J.F., Hvas C.L. (2020). Faecal microbiota transplantation for recurrent Clostridioides difficile infection: An updated systematic review and meta-analysis. EClinicalMedicine.

[70] Kim K.O., Gluck M. (2019). Fecal Microbiota Transplantation: An Update on Clinical Practice. Clin. Endosc..

[71] Freitag T.L., Hartikainen A., Jouhten H., Sahl C., Meri S., Anttila V.-J., Mattila E., Arkkila P., Jalanka J., Satokari R. (2019). Minor Effect of Antibiotic Pre-treatment on the Engraftment of Donor Microbiota in Fecal Transplantation in Mice. Front. Microbiol..

[72] Oliphant K., Cochrane K., Schroeter K., Daigneault M.C., Yen S., Verdu E.F., Allen-Vercoe E. (2020). Effects of Antibiotic Pretreatment of an Ulcerative Colitis-Derived Fecal Microbial Community on the Integration of Therapeutic Bacteria In Vitro. mSystems.

[73] Ji S.K., Yan H., Jiang T., Guo C.Y., Liu J.J., Dong S.Z., Yang K.L., Wang Y.J., Cao Z.J., Li S.L. (2017). Preparing the Gut with Antibiotics Enhances Gut Microbiota Reprogramming Efficiency by Promoting Xenomicrobiota Colonization. Front. Microbiol..

