# Supplementary material for: Repeated Fecal Microbial Transplantations and Antibiotic Pre-Treatment Are Linked to Improved Clinical Response and Remission in Inflammatory Bowel Disease: A Systematic Review and Pooled Proportion Meta-Analysis
Source: J Clin Med. 2021 Mar 1;10(5):959. doi: 10.3390/jcm10050959 (PMC7957789; doi:10.3390/jcm10050959)
Supplement: Supplementary file 1 [file jcm-10-00959-s001.zip › Supplemental Info/Supplemental Information-Pretreatment.docx]

Supplemental Table S1: Full-text search strategy of included databases.

| **Database** | **Search Strategy** |
| --- | --- |
| **MEDLINE**  **Ovid MEDLINE(R) ALL** 1946 to January 28, 2021 | 1. Fecal Microbiota Transplantation/  2. FMT.ti,ab.  3. feces infusion*.mp.  4. donor feces.mp.  5. (stool adj2 transplant*).mp.  6. f?ecal transfusion*.mp.  7. f?ecal bacteriotherap*.mp.  8. (f?ecal adj3 transplant*).mp.  9. or/1-8  10. exp Inflammatory Bowel Diseases/ or inflammatory bowel disease*.mp. 11. Crohn*.mp.  12. ulcerative colitis.mp.  13. IBD.ti,ab.  14. indeterminate colitis.mp.  15. or/10-14  16. 9 and 15  17. Animals/ or (veterinary or rabbit or rabbits or animal or animals or mouse or mice or rodent or rodents or rat or rats or pig or pigs or porcine or horse* or equine or cow or cows or bovine or goat or goats or sheep or ovine or canine or dog or dogs or feline or cat or cats or zebrafish).ti.  18. Humans/  19. 17 not (17 and 18)  20. 16 not 19 |
| **Embase**  **Ovid Embase** 1974 to 2021 January 28 | 1. fecal microbiota transplantation/  2. FMT.ti,ab.  3. feces infusion*.mp.  4. donor feces.mp.  5. (stool adj2 transplant*).mp.  6. f?ecal transfusion*.mp.  7. f?ecal bacteriotherap*.mp.  8. (f?ecal adj3 transplant*).mp.  9. or/1-8  10. exp inflammatory bowel disease/ or inflammatory bowel disease*.mp. 11. Crohn*.mp.  12. ulcerative colitis.mp.  13. IBD.ti,ab.  14. indeterminate colitis.mp.  15. or/10-14  16. 9 and 15  17. animal/ or (veterinary or rabbit or rabbits or animal or animals or mouse or mice or rodent or rodents or rat or rats or pig or pigs or porcine or horse* or equine or cow or cows or bovine or goat or goats or sheep or ovine or canine or dog or dogs or feline or cat or cats or zebrafish).ti.  18. human/  19. 17 not (17 and 18)  20. 16 not 19 |
| **Scopus** | TITLE-ABS-KEY ( fmt  OR  "feces infusion*"  OR  "donor feces"  OR  ( stool  W/2  transplant* )  OR  "fecal bacteriotherap*"  OR  "faecal bacteriotherap*"  OR  ( fecal  W/3  transplant* )  OR  ( faecal  W/3  transplant* ) )  AND  TITLE-ABS-KEY ( "inflammatory bowel disease*"  OR  crohn*  OR  "ulcerative colitis"  OR  ibd  OR  "indeterminate colitis" )  AND NOT  TITLE ( veterinary  OR  rabbit  OR  rabbits  OR  animal  OR  animals  OR  mouse  OR  mice  OR  rodent  OR  rodents  OR  rat  OR  rats  OR  pig  OR  pigs  OR  porcine  OR  horse*  OR  equine  OR  cow  OR  cows  OR  bovine  OR  goat  OR  goats  OR  sheep  OR  ovine  OR  canine  OR  dog  OR  dogs  OR  feline  OR  cat  OR  cats  OR  zebrafish ) |
| **Web of Science Core Collection** | #1  TS= (fmt OR "feces infusion*" OR "donor feces" OR ( stool NEAR/2 transplant* ) OR "fecal bacteriotherap*" OR "faecal bacteriotherap*" OR ( fecal NEAR/3 transplant* ) OR ( faecal W/3 transplant* )) AND TS= ("inflammatory bowel disease*" OR crohn* OR "ulcerative colitis" OR ibd OR "indeterminate colitis")  #2  TI=(veterinary OR rabbit OR rabbits OR animal OR animals OR mouse OR mice OR rodent OR rodents OR rat OR rats OR pig OR pigs OR porcine OR horse* OR equine OR cow OR cows OR bovine OR goat OR goats OR sheep OR ovine OR canine OR dog OR dogs OR feline OR cat OR cats OR zebrafish)  #3  #1 NOT #2 |
| **Cochrane Library**  via Wiley | #1 MeSH descriptor: [Fecal Microbiota Transplantation] this term only  #2 FMT:ti,ab  #3 feces infusion*  #4 donor feces  #5 stool NEAR/2 transplant*  #6 f?ecal next transfusion*  #7 f?ecal next bacteriotherap*  #8 f?ecal NEAR/3 transplant*  #9 #1 OR #2 OR #3 OR #4 OR #5 OR #6 OR #7 OR #8  #10 MeSH descriptor: [Inflammatory Bowel Diseases] explode all trees  #11 inflammatory next bowel next disease*  #12 Crohn*  #13 ulcerative colitis  #14 IBD:ti,ab  #15 indeterminate colitis  #16 #10 OR #11 OR #12 OR #13 OR #14 OR #15  #17 #9 AND #16  #18 (veterinary OR rabbit OR rabbits OR animal OR animals OR mouse OR mice OR rodent OR rodents OR rat OR rats OR pig OR pigs OR porcine OR horse* OR equine OR cow OR cows OR bovine OR goat OR goats OR sheep OR ovine OR canine OR dog OR dogs OR feline OR cat OR cats OR zebrafish):ti  #19 #17 NOT #18 |
| **Google Scholar** | ("fecal transplant" OR "fecal transfusion" OR FMT OR "feces infusion" OR "donor feces" OR "stool transplant") AND ("inflammatory bowel disease" OR Crohn's OR "ulcerative colitis" OR "indeterminate colitis") |

**Supplemental Table S2.** Newcastle-Ottawa scale for assessing risk of bias for included cohort studies.

a-Inadequate microbiome description.

| **Quality assessment scale** | **Accepted criteria** | **Chen 2020** | **Chen 2020** | **Cold 2019** | **Cui 2015** | **Damman 2015** | **Ishikawa 2017** | **Jacob 2017** | **Kump 2017** | **Mizuno 2017** | **Nishida 2017** | **Okahara 2020** | Schierova 2020 | **Sood 2019** | **Sood 2020** | **Uygun 2017** | **Vaughn 2016** | **Vermeire 2016** | **Wei 2015** | **Zhang 2016** | **Wang 2020** | |  |
| --- | --- | --- | --- | --- | --- | --- | --- | --- | --- | --- | --- | --- | --- | --- | --- | --- | --- | --- | --- | --- | --- | --- | --- |
| **Selection** | | | | | | | | | | | | | | | | | | | | | |  | |
| Representativeness of the exposed cohort | Representative of average IBD adults | * | * | * | * | * | * | * | * | * | * | * | * | * | * | * | * | * | * | * | * | |  |
| Ascertainment of FMT | Secure records | * | * | * | * | * | * | * | * | * | * | * | * | * | * | * | * | * | * | * | * | |  |
| Demonstration that outcome of interest was not present at start of study | Evidence of no prior FMT exposure | * | * | * | * | * | * | * | * | * | * | * | * | * | * | * | * | * | * | * | * | |  |
| **Comparability** | | | | | | | | | | | | | | | | | | | | | |  | |
| Comparability of cohorts on the basis of the design or analysis | Described FMT regiment and delivery, current medications, disease severity, microbiome analysis | a | * | * | a | * | * | * | * | * | * | * | * | a | a | a | * | * | a | a | a | |  |
| **Outcome** | | | | | | | | | | | | | | | | | | | | | |  | |
| Assessment of outcome | Pre-defined cut-off points for response and remission | * | * | * | * | * | * | * | * | * | * | * | * | * | - | * | * | * | * | * | * | |  |
| Was follow-up long enough for outcomes to occur? | 3 months | * | * | * | * | - | - | - | * | * | - | - | * | * | * | * | - | - | - | * | - | |  |
| Adequacy of follow-up of cohorts | Follow up of complete cohort or appropriate characterization of dropouts | * | * | * | * | * | * | * | * | * | * | * | * | * | * | * | * | * | * | * | * | |  |
| **Total** (Max = 7) | | 6 | 7 | 7 | 6 | 6 | 6 | 6 | 7 | 6 | 6 | 6 | 7 | 6 | 5 | 6 | 6 | 6 | 5 | 6 | 5 | |  |

a-Inadequate microbiome description

**Supplemental Tables S3.** Cochrane risk of bias assessment for included randomized trials.

- Please see attached files that can be merged or kept independently as needed.
